# Clinical mentoring to improve quality of care provided at three NIM-ART facilities: A mixed methods study

**DOI:** 10.4102/phcfm.v10i1.1579

**Published:** 2018-06-14

**Authors:** Chris A. Visser, Jacqueline E. Wolvaardt, David Cameron, Gert J.O. Marincowitz

**Affiliations:** 1 School of Health Systems and Public Health, University of Pretoria, South Africa; 2 Foundation for Professional Development, Pretoria, South Africa; 3 Department of Family Medicine and Primary Health Care, University of Limpopo, South Africa

## Abstract

**Background:**

The South African Department of Health implemented the nurse-initiated management of antiretroviral treatment (NIM-ART) programme as a policy to decentralise services. Increasing access to ART through nurse initiation results in significant consequences.

**Aim:**

This study evaluated the quality of care provided, the barriers to the effective rollout of antiretroviral services and the role of a clinical mentor.

**Setting:**

The study was conducted at three NIM-ART facilities in South Africa. One clinic provided a high standard of care, one had a high defaulter rate, and at the third clinic, treatment failures were missed, and routine bloods were not collected.

**Methods:**

A mixed methods study design was used. Data were collected using patient satisfaction surveys, review of clinical records, facility audits, focus group interviews, field notes and a reflection diary.

**Results:**

NIM-ART nurses prescribed rationally and followed antiretroviral guidelines. Mortality rates and loss to follow-up rates were lower than those at the surrounding hospitals, and 91.1% of nurse-monitored patients had an undetectable viral load after a year. The quality of care provided was comparable to doctor-monitored care. The facility audits found recurrent shortages of essential drugs. Patients indicated a high level of satisfaction. Salary challenges, excessive workload, a lack of trained nurses and infrastructural barriers were identified as barriers. On-going mentoring and support by a clinical mentor strengthened each of the facilities, facilitated quality improvement and stimulated health workers to address constraints.

**Conclusion:**

Clinical mentors are the key to addressing institutional treatment barriers and ensuring quality of patient care.

## Introduction

The South African Department of Health implemented the policy of nurse-initiated management of antiretroviral treatment (NIM-ART) in 2010. This policy aims to decentralise antiretroviral (ARV) services to primary health care facilities. It entails task shifting and is intended to provide ARV services to the individuals who require them most.^1^ A prospective cohort study found that although increasing numbers of patients had started ART after the national rollout in 2004, many patients died before they could access ART. The study concluded that patient outcomes could be improved by decentralisation of treatment services, fast-tracking the most immune-deficient patients and improving access, especially for men.^2^

Nursing staff at decentralised primary health care facilities are often overburdened, with long queues, demanding working hours and a high demand for primary health care services. A qualitative study by Stein, which evaluated the health system effect of the NIM-ART policy, revealed that increasing access to ART through nurse initiation results in significant knock-on effects in terms of training and support needs, workload and capacity constraints, logistical and infrastructural challenges, and shifts in the working and referral relationships between health care workers.^3^ A review of the impact of human immunodeficiency virus (HIV) and acquired immunodeficiency syndrome (AIDS) programmes on health worker retention, showed critical health worker shortages as a major barrier to scale-up of comprehensive HIV and AIDS services, especially in rural areas. HIV and AIDS programmes create greater demand for health care workers. In response, there have been efforts to recruit additional staff, including new cadres such as lay counsellors and community health workers. Depending on the country involved, the additional staff may be from the existing pool of health care workers or could be from outside the system.^4^

Long et al. found that nurse-monitored ARV care was not inferior to doctor-monitored ARV care. The 12-month outcomes of stable patients on ART who were down referred to a primary health clinic were found to be comparable to, or better than, the outcomes of similar patients who were maintained at a hospital-based ARV clinic. The study found that the down referral site experienced less death and loss to follow-up than the treatment initiation site.^5^ A cluster, randomised study found HIV and AIDS care provided by nurses to be as effective and safe as doctor-provided care. Furthermore, the study found that nurse centred care for HIV patients provided significant health benefits such as the earlier detection of tuberculosis as well as improved treatment adherence. The survival rates and viral suppression observed in the study were comparable to the outcomes of doctor-provided care.^6^

Sanne et al. conducted a randomised non-inferiority trial at two South African primary care clinics. HIV-positive individuals with a CD4 (cluster of differentiation 4) cell count of less than 350 cells/µL or patients with who stage 3 or 4 disease were randomly assigned to either nurse-monitored, or doctor-monitored ART care. The primary objective was a combined end point of treatment limiting events, mortality, treatment failure, the development of adverse side effects to ARV drugs and adherence to treatment. After a median follow-up of 120 weeks, the end points were found to be similar in the nurse and doctor groups, supporting the view that nurse-monitored ARV care is comparable to doctor-monitored care.^7^

In rural and remote areas, health services are often rendered under demanding circumstances. The Greater Tzaneen municipality is part of the rural Mopani district in South Africa, to which the national government has allocated the status of a ‘priority district’ on the basis of the region’s underdevelopment, its prevailing high rates of poverty and unemployment, as well as poor quality of education and health care services.^8^

The critical shortage of human resources for health in the public sector, in particular medical doctors, is a major barrier to achieving universal access to HIV care and treatment in rural areas in South Africa. Task shifting through NIM-ART is a solution to expanding access to ART, and although the evidence for non-physician provided ART in South Africa is limited, the available evidence suggests that NIM-ART could be a potentially effective, sustainable and widely acceptable approach to combat HIV and its devastating effects.^9^ Both clinical mentoring and supportive supervision are needed to build the health service delivery systems needed for NIM-ART.^10^ Although supportive supervision focuses mainly on the management of the health facility (e.g. drug supply and equipment), the goals of clinical mentoring are to ‘improve patients’ clinical outcomes, support decentralisation of health care delivery with high quality of care, strengthen problem solving and clinical decision making skills of the health care provider, and build the capacity of providers to manage or refer unfamiliar or complicated cases, as appropriate’.^10^

The aim of this study was, firstly, to evaluate the quality of care provided at three selected NIM-ART facilities in the Greater Tzaneen sub-district of Limpopo province and, secondly, to explore the effects of clinical mentoring and support on improving the quality of care.

## Methods

A mixed methods study that used concurrent quantitative and qualitative research methods was conducted.^11^

The study was conducted at three NIM-ART facilities in the Greater Tzaneen municipality for a six-month period. The three clinics who met the selection criteria were purposively sampled. The three NIM-ART facilities were chosen for their uniqueness and individual characteristics. One clinic was an early adopter of the NIM-ART programme and provided a high standard of care (Clinic A), one had a high defaulter rate (Clinic B), and at the last facility, treatment failures were often missed and routine bloods were not collected (Clinic C). The selection of the clinics was based on routine performance data that were collected monthly.

The researcher had already provided clinical mentoring to the three NIM-ART facilities prior to the commencement of the study and was engaged in quality improvement activities with these facilities during the course of the study. A comparison of data was performed in three phases per clinic. The first phase served as the pilot for the study and the testing of the tools such as a patient file checklist and the patient satisfaction questionnaire. The second phase was characterised by the mentor led educational intervention, the workplace-based learning, mentor observation and feedback of individual consultations and reflections of practice that took place during the clinic meetings. Flowcharts that simplified the more complex national treatment guidelines and clinic specific improvement plans were developed by the researcher and the clinical staff in this phase. The document review and facility audit were also performed by the researcher and the clinic staff in this second phase in order to identify opportunities for improvement. In the third phase, we conducted patient satisfaction surveys and focus group interviews in addition to the on-going mentor led clinical support activities, document reviews and facility audits.

Data were collected from document reviews, facility audits, patient satisfaction surveys, focus group interviews with staff, field notes and a reflection diary.

### Quantitative research methods and analysis

Data sources used in the document review component of the study included patients’ clinical records, ARV registers and drug stock levels.

The national ARV guidelines and the NIM-ART policies were used as standards for the comparison of the data in the document review of the clinical records. Deficits in performance were identified by the clinical mentor by using a patient file audit tool that included viral loads done, CD4 collected, viral load supressed, absence of serious adverse events, correct ARV regimen prescribed, remaining in care and no death on treatment. Quality improvement interventions such as ensuring that viral loads were done were subsequently instituted.

In order to prevent selection bias, every third clinical record was selected by the data capturer at each of the NIM-ART facilities and kept for audit. A total of 537 clinical records were audited. This number is higher than what was suggested by the statistician (*n* = 332) as the staff did facility audits as part of their workplace-based learning. Performance indicators included among other things: the collection of monitoring bloods, rational prescribing, evidence of treatment failure (defined as viral load > 1000 copies/mL) and loss to follow-up (defined as having missed a scheduled clinic visit by 90 days or more) or death.

The facility audit checklist and the patient satisfaction survey questionnaire were developed from core standards for Primary Health Care (PHC) clinics.^12^ The checklist was completed by the facility manager and reviewed by the clinical mentor.

A patient satisfaction survey was completed at each of the three NIM-ART facilities in the final phase of the study. The sample size for the patient survey (*n* = 332) was determined by a statistician and was based on the relative patient population at each of the facilities. Patients who were enrolled at the clinics were asked to rate the facility’s accessibility, waiting period for results, health workers’ attitude and care, the facility’s structure, confidentiality and whether they would recommend the NIM-ART facility to others. The rating was on a scale of: 1 (poor) – 5 (excellent). Lay counsellors served as research assistants and administered the questionnaires. Finally, more (*n* = 354) than the minimum number were sampled in order to achieve the required sample size.

The quantitative data were entered into Excel spreadsheets, and descriptive and inferential statistical analysis was performed using STATA software. Associations between categorical variables were determined using chi-square analysis. A *p*-value of < 0.05 was considered statistically significant.

### Qualitative research methods and analysis

At each clinic, action learning groups were created from the facility staff. These action learning groups consisted of the clinical mentor, the clinic manager, professional nurses who were practicing as NIM-ART nurses, professional nurses who were not yet part of the NIM-ART programme, lay counsellors and data capturers. The clinical mentor was the only doctor at all three clinics. At the time, there was no non-governmental organisational support so all the participants were employed by the government.

Nine focus group interviews were conducted among the three NIM-ART facilities (three cycles per clinic) to evaluate staff perceptions of, and attitudes towards, the NIM-ART programme. All the clinic staff participated in the focus group interviews, and the composition remained constant over all three interviews. Categories of health workers other than nurses were included to gain an enriched understanding of the perceptions of all members of the diverse NIM-ART team. The focus group interviews were conducted by the clinical mentor who has experience and training in conducting focus group interviews.

During the focus group interviews, participants were specifically asked to identify the root causes for resistance to the NIM-ART programme, as well as the barriers preventing the programme’s effective implementation. Demographic information was obtained prior to the focus group interviews.

The qualitative data were analysed with the use of ATLAS.ti software. An inductive approach was followed in the coding, and the emerging themes were identified. Credibility of the qualitative data was achieved through triangulation by using a variety of data collection methods such as the patient satisfaction survey, document review and the facility audit. Transferability of the data was achieved by thick description of the qualitative findings, whereas dependability was achieved by collecting data until the data were saturated. Confirmability was achieved through recording the interviews, the compilation of field notes during the interviews and keeping a research diary as part of the audit trail.

## Ethical considerations

Ethical approval was obtained from the Faculty of Health Sciences Research Ethics Committee of the University of Pretoria (clearance certificate 215/2011). Permission was obtained from the head of department of the Limpopo province Department of Health and Social Development, as well as from the operational managers of the three NIM-ART facilities, before the commencement of the study. Participation was voluntary and anonymous (patient questionnaire) or confidential (focus group interviews).

## Results

### Patient satisfaction survey

**FIGURE 1:**
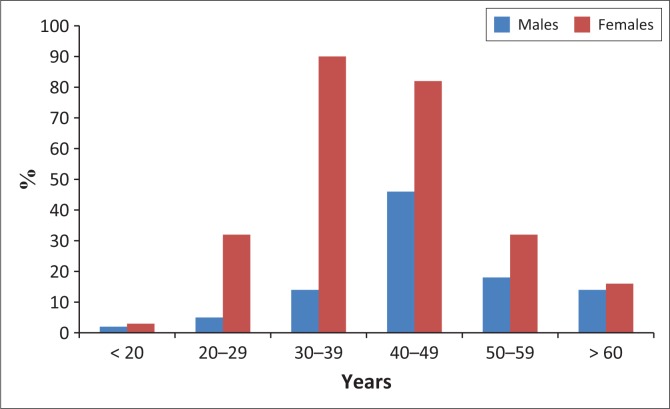
Age and gender distribution of participants of the patient satisfaction survey.

A sample of 354 patients were surveyed, with 62 (Clinic A), 233 (Clinic B) and 59 (Clinic C) patients being surveyed, respectively (Figure 1).

The results of the patient satisfaction survey revealed a high level of patient satisfaction and no difference in patients’ opinions between the three facilities. Almost half indicated that they would be highly likely to recommend their NIM-ART facility to others; 106 (29.9%) would be likely to recommend it, whereas only 19 (5.4%) indicated that they would not recommend it (Table 1).

**TABLE 1:** Patient satisfaction survey ratings (*n* = 354).

Standard	Excellent		Good		Neutral		Fair		Poor	
	*n*	%	*n*	%	*n*	%	*n*	%	*n*	%
**Access**										
Ability to access care	183	51.7	84	23.7	45	12.7	14	4.0	28	7.9
Clinic’s opening hours	161	45.5	109	30.8	38	10.7	25	7.1	21	5.9
Convenience of clinic location	167	47.2	96	27.1	51	14.4	13	3.7	27	7.6
**Waiting**										
Time waited before seeing a health worker	122	34.5	118	33.3	49	13.8	30	8.5	35	9.9
Waiting for test results	174	49.1	113	31.9	25	7.1	19	5.4	23	6.5
**Staff**										
Listens to you	180	50.9	106	29.9	39	11.0	11	3.1	18	5.1
Takes enough time with you	179	50.6	106	29.9	30	8.5	21	5.9	18	5.1
Explains what you want to know	173	48.9	107	30.2	41	11.6	13	3.7	20	5.6
Gives you good advice and treatment	197	55.6	99	28.0	32	9.0	12	3.4	14	4.0
Friendly and helpful to you	200	56.5	94	26.6	29	8.2	10	2.8	21	5.9
**Facility**										
Neat and clean building	168	47.5	125	35.3	27	7.6	15	4.2	19	5.4
Ease of finding where to go	172	48.6	116	32.7	42	11.9	12	3.4	12	3.4
Comfort and safety while waiting	159	44.9	125	35.3	39	11.0	6	1.7	25	7.1
Privacy	173	48.9	102	28.8	26	7.3	23	6.5	30	8.5
Keeping my personal information private	190	53.7	92	26.0	35	9.9	16	4.5	21	5.9
**Recommendation**										
How likely am I to refer others to this clinic?	176	49.7	106	29.9	42	11.9	11	3.1	19	5.4

The two worst-rated aspects (fair and poor combined) were, time waited before seeing a health worker (18.4%) and privacy (15%). Waiting for test results and ability to access care were the third worst-rated aspects and were equally scored at 11.9%.

No statistically significant differences were found in the responses given when comparing male and female participants, or when comparing patients from different NIM-ART facilities. Neither did comparing the responses of younger patients (< 35 years) to older (≥ 35 years) patients reveal any differences. However, patients who had started ART at hospital and were subsequently down referred to NIM-ART facilities tended to rate the performance of clinics lower (rating < 3/5).

### Document review

Out of the 537 adult and children files reviewed, it was evident that NIM-ART nurses followed the national ARV policies when initiating and maintaining ART (measured by eligibility for initiation, correct prescriptions, bloods taken at the correct time, completion of patient records, correct management of side effects and patient adherence to ART with a suppressed viral load).

No ineligible patients were started on ART. Only one (0.9%) of the audited patient files contained an incorrect prescription. The combination of stavudine and zidovudine was wrongly prescribed and lamivudine was omitted. This patient’s prescription was subsequently corrected by the clinical mentor.

Eleven patients (2.0%) developed adverse ARV drug-related side effects (based on the guidelines at the time).^1^ Two patients (0.4%) developed severe side effects, whereas the remaining nine patients (1.7%) had either minor side effects of ARV drugs or developed lipodystrophy as a result of long-term stavudine therapy. Of the two patients with severe side effects, one patient (0.9%) developed Steven–Johnson syndrome because of nevirapine toxicity and the other (0.9%) developed hepatotoxicity while taking ART and anti-tuberculosis treatment simultaneously. Both these side effects were identified and both patients were promptly referred. The treatment regime of the two patients with severe side effects was subsequently changed by the clinical mentor.

The less severe side effects included minor skin rashes, diarrhoea, headaches, as well as the development of mild peripheral neuropathy and insomnia. Three patients (0.6%) who developed lipodystrophy were switched to an alternative regimen. All minor side effects were appropriately managed according to treatment protocols.

On average, patients taking treatment at these facilities gained 3.7 kg in the first year on treatment.

Of the files audited, 91.1% of patients had an undetectable viral load (< 40 viral copies/mL) after 1 year of nurse-monitored care. The treatment failure rates at the three study sites (Table 2) were below the treatment failure rate experienced at hospitals in the Greater Tzaneen municipal area at the time of the study.

**TABLE 2:** Comparison of clinic performance and selected indicators

Variable	Average baseline CD4 count (cells/μL)	End-of-study CD4 count after 12 months (cells/μL)	Baseline lost to follow-up (%)	End-of-study lost to follow-up (%)	Treatment failure rates (%)	Highest recorded monthly mortality	
						*n*	%
Clinic A	44	146	4.1	0.3	2.6	4	0.5
Clinic B	51	113	7.3	0	1.1	1	0.5
Clinic C	83	171	2.1	0	1.6	2	0.8

In this study, the mortality rate of patients receiving care at the three NIM-ART facilities remained below 1%. Most of the 15 patients (*n* = 11) who died had arrived at the facilities with advanced HIV or had severe opportunistic co-infections.

### Facility audit

At the start of this study, only one NIM-ART facility with three NIM-ART-trained nurses provided paediatric ARV care. However, at the end of the study, all NIM-ART nurses at the three study sites initiated and maintained children on ART.

Routine blood investigations had not been performed on 17.7% – 41.4% of adult patients at the start of the study. The reasons for the non-collection of routine bloods were a high workload, a lack of trained NIM-ART nurses and the unavailability of specimen bottles. Task shifting was implemented by allocating routine duties previously performed by NIM-ART nurses to nursing assistants and through the involvement of other cadres of health workers in the programme. By the end of the study, blood investigations were performed for almost all patients as a result of the implementation of task shifting, with routine blood investigations not being performed in 1.6% – 3.8% of instances, depending on the facility.

Of concern was the erratic drug supply, especially the regular stock out of essential ARV drugs at the facilities, during the course of the study. In particular, tenofovir was regularly unavailable and NIM-ART nurses were compelled to change patients to an alternative ARV drug regimen. Isoniazid was often in short supply, and the facility audit showed that there was no stock at any of the three facilities for two months.

### Focus group interviews

Table 3 shows the demographic characteristics of the participants in the focus group interviews.

**TABLE 3:** Demographic characteristics of participants in the focus groups.

NIM-ART facility	Participant	Occupation	Gender	Years worked as health worker	Years worked with HIV and AIDS patients	Training courses attended
Clinic A	1	Data capturer	Male	< 1	< 1	Data capturing
	2	Nurse	Male	5–10	5–10	NIM-ART; VCT
	3	Counsellor	Female	< 1	< 1	Counselling
	4	Nurse	Female	5–10	5–10	NIM-ART; VCT
	5	Nurse	Female	15–20	5–10	VCT
Clinic B	1	Counsellor	Female	5–10	5–10	Counselling
	2	Counsellor	Female	1–5	1–5	Counselling
	3	Nurse	Female	10–15	5–10	NIM-ART; VCT
	4	Data capturer	Female	< 1	< 1	Data capturing
	5	Nurse	Female	< 1	< 1	NIM-ART; VCT
Clinic C	1	Nurse	Female	> 20	1–5	VCT
	2	Nurse	Female	> 20	1–5	NIM-ART; VCT
	3	Nurse	Female	15–20	1–5	NIM-ART; VCT
	4	Counsellor	Male	1–5	1–5	Counselling
	5	Data capturer	Female	1–5	1–5	Data capturing

VCT, voluntary counselling and testing.

Note: This counselling service is offered to people so that they are able to make an informed decision about whether to be tested for HIV.

Emergent themes from the focus groups were salary challenges, excessive workload, a lack of trained NIM-ART nurses and infrastructural barriers.

#### Salary challenges

A lack of incentives and non-payment of lay counsellors’ stipends emerged as a challenge:

‘As a lay counsellor I work too much, but monthly we sometimes do not get our stipend. For us as counsellors it is discouraging to work with the people and you see the improvement of the people, but at the end of the day you don’t get enough salary or stipend.’ (Participant B2, female, counsellor)

#### Excessive workload

Excessive workload was one of the major reasons why health workers might be resistant to participate in the NIM-ART programme:

‘If Monday comes you would think twice if I should come to work on not, because I’m going to be stressed during the whole day… few nurses are trained on NIM-ART. If a NIM-ART nurse can only attend to NIM-ART for the whole week and not implement another PHC package, it would be easier.’ (Participant C3, female, nurse)

#### Lack of nurse-initiated management of antiretroviral treatment-trained nurses

A lack of trained nurses prevented the effective implementation of the NIM-ART programme:

‘Few nurses are trained in NIM-ART. This is hampering the effective implementation of the NIM-ART programme’. (Participant A4, female, nurse)

#### Infrastructural barriers

Participants were enthusiastic about the NIM-ART programme and its benefits. Focus group participants named drug shortages, difficulties in obtaining laboratory results and a lack of phones at the clinics as the major infrastructural barriers to the successful implementation of the programme.

**Drug shortages were noted as a major barrier:**

‘Now we are running short of the drugs’ and patients are now confused. … On their previous regimen they only needed to take treatment once a day, now they have to take it twice a day. We are going to have many defaulters, because patients don’t like it when we change their treatment.’ (Participant B5, female, nurse)

Critical medicine shortages created frustrations for nurses and patients:

‘You find us swopping the treatment of clients unnecessarily because of drugs shortages. This is a barrier to us’. (Participant A2, male, nurse)

**Difficulty in obtaining laboratory results:** Inadequate laboratory services and prolonged turnaround time for blood investigations were another barrier:

‘… the results come in late. Patients come, and you find that the result is not yet back from the laboratory’ (Participant A1, male, data capturer)

‘… and we are having a problem where you see you have taken CD4 bloods from a patient then the results are not coming back. When you call lab, they say they have not received the result.’ (Participant A1, male, data capturer)

**Lack of phones:** A lack of phones at the clinics was stated as a further barrier:

‘We are unable to communicate with the lab to obtain results. Sometimes we want to fast track our clients. We don’t have access to phones to talk to our clients. Communication is a problem.’ (Participant C5, female, counsellor)

‘… and ‘I don’t know how to trace patients. We don’t have a cell phone to contact them or any car to go and find them’. (Participant C5, female, counsellor)

### Role of the clinical mentor

The reflective process followed by the clinical mentor during the study provided insight into ways in which a clinical mentor could strengthen the NIM-ART programme. Initially, the skills and knowledge of all team members were identified during the facility audit, and a specific mentoring plan was developed for each of the facilities. Information was shared about best practices. An iterative approach was followed to address the problem of resistance among health workers to participate in NIM-ART care.

The practitioner researcher performed self-reflected practice by keeping a research diary and also shared research skills such as learning while doing, with the health workers at the individual primary care facilities. On-going mentoring and support by the practitioner researcher strengthened each of the facilities, facilitated quality improvement and provided confidence for more health workers to become actively involved.

Positive responses about the role of a clinical mentor were received during the focus group interviews. A participant remarked about the value of a clinical mentor:

‘We want to thank you for opening our eyes, because most of the work we were ignoring then, but after this programme we managed to come back to our senses and concentrate on our clients and now we know are clients are well and we are doing the correct thing.’ (Participant C3, female, nurse)

Involving all facility staff in decision making, good communication and regular feedback to all stakeholders contributed to strengthening the programme. By involving all facility staff in the research process, the research process in itself resulted in improved performance.

## Discussion

In this study, the physical infrastructure in which care is delivered appears to be uniform with patients at the three sites reporting high levels of satisfaction with the service and no differences in satisfaction between the sites. The only significant difference was found in those who were down referred from a hospital and who were less satisfied with the clinic’s care. This finding might be explained by the perceived loyalty these patients developed towards the facility where their care was initiated, despite the intention of providing care closer to home.

This study confirms the results of previous studies, namely that nurse-monitored care was not inferior to doctor-monitored care^5^ and that the outcomes of patients attending primary care clinics were similar to those attending hospital clinics with similar rates of survival, viral load suppression, adherence, treatment failure and adverse events.^6,7^ The use of standard treatment guidelines by all nurses contributed to the uniformity of care at all three sites. Over 90% of patients had an undetectable viral load after one year of treatment with lower rates of treatment failure, mortality and loss to follow-up than those observed in the surrounding hospitals. The provision of paediatric care was improved over the course of the study as the two sites who did not provide paediatric care at the start were supported and mentored until they too provided care. Task shifting and clinical mentoring resulted in substantial improvements in the collection of routine bloods, with the worst performing clinic improving from over 40% non-collection to less than 4%. The expansion of paediatric care and the improvement of the collection of routine bloods are examples of the benefits of clinical mentoring.^10^

Policy-makers usually prioritise three dimensions of quality: efficiency, effectiveness and safety.^13^ These priorities are reflected in developing policies, treatment guidelines and setting standards. This study showed the benefit of using treatment guidelines and applying standards in the workplace, and also highlighted the barriers to preventing the programme’s effective implementation, an aspect not explored in previous studies.^5,6,7^ It is policy-makers alone who can solve the problems related to salaries for counsellors, the excessive workload, the lack of trained NIM-ART nurses and infrastructure barriers.

It is recommended that policy-makers focus on addressing those problems beyond the scope of clinical mentors, strengthening human resources, improving supply chain management and the turnaround times for routine blood investigations.

## Limitations

The patient survey could only be conducted among the patients who were retained in care and not lost to follow-up or who died. This limitation, therefore, results in a selection bias.

## Conclusion

This study confirmed that NIM-ART provided care is possible and effective even within a poorly performing district and in spite of numerous systems challenges at PHC facilities.

The quality of care provided and patient outcomes at the three NIM-ART facilities met national ARV service standards, with a high level of care being maintained at each of the nurse-monitored facilities, in spite of the complex health system challenges. Some of the system challenges can be overcome by clinical mentoring that takes not only individual patient care into consideration but also a mentoring approach that uses research to systematically address barriers to care.

This research project not only provided insight into the performance at the NIM-ART facilities and the barriers preventing the successful implementation of the NIM-ART programme, but also enhanced the quality of ARV patient care at each of NIM-ART facilities.
